# Impaired sensitivity to thyroid hormones is associated with ASCVD risk factors in Beijing in euthyroid population: a cross-sectional study

**DOI:** 10.3389/fendo.2025.1602202

**Published:** 2025-07-17

**Authors:** Lintao Shi, Jianjun Wang, Li Yang, Weichao Zhao, Min Zhang, Aiyun Yu, Lingfang Ni, Yu Liu, Haiying Jia

**Affiliations:** ^1^ Department of Special Service Health Management, Ninth Medical Center of Chinese People’s Liberation Army (PLA) General Hospital, Beijing, China; ^2^ Department of Endocrinology, Ninth Medical Center of Chinese People’s Liberation Army (PLA) General Hospital, Beijing, China; ^3^ Department of Respiratory, Ninth Medical Center of Chinese People’s Liberation Army (PLA) General Hospital, Beijing, China

**Keywords:** thyroid hormones resistance, atherosclerotic cardiovascular disease, dyslipidemia, uric acid, blood pressure

## Abstract

**Background:**

This review aims to investigate the relationship between impaired sensitivity to thyroid hormones and ASCVD risk factors in the euthyroid population.

**Methods:**

A cross-sectional study including 7,895 euthyroid subjects aged ≥18 years old was conducted. Height, body weight, blood pressure (BP) were measured, and serum concentrations of lipids, fasting blood glucose, uric acid, free thyroxine (FT4), free triiodothyronine (FT3), and thyrotropin (TSH) were assayed. Thyroid hormone resistance was calculated by Thyroid-stimulating hormone (TSHI), Thyroid Feedback Quantile-based Index (TFQI), and Free Triiodothyronine/Free thyroxine (FT3/FT4), which were calculated based on FT3, FT4, and TSH.

**Results:**

We are divided into four groups according to the TFQI quartile. Age, BMI, BP, HDL-C, TC, TG, FBG, UA were statistically significant between the four groups. F3/F4 showed statistical significance in populations with high UA, high FBG, and high TG; TFQI was positively correlated with BP, TG, FBG, UA and TyG, SBP, FBG, UA and TyG, FT3/FT4 was negatively correlated with DBP, SBP, TC, TG and UA.

**Conclusion:**

Impaired sensitivity to thyroid hormone in euthyroid population is associated with ASCVD risk factors. These findings are potentially useful for understanding the interaction between thyroid hormone sensitivity and ASCVD risk factors in euthyroid population.

## 1 Introduction

Thyroid hormones are key regulators of energy metabolism, and a close association with metabolic disorders has been demonstrated, even when thyroid hormones are within the normal range (1). Patients with overt and subclinical hypothyroidism (SCH) have a higher risk of cardiovascular disease (2). SCH may exacerbate several metabolic abnormalities, such as hypertension, atherosclerosis, dyslipidemia, and abnormal endothelial function (3).

Euthyroidism was defined as having no thyroid dysfunction, including clinical and subclinical hypo- and hyperthyroidism. Most subjects at risk for cardiovascular disease are euthyroid. Impaired thyroid hormone sensitivity has been associated with metabolic disorders such as diabetes and metabolic syndrome, which are known risk factors for cardiovascular diseases (CVD) (4, 5). Laclaustra et al. first proposed the thyroid feedback quantile-based index (TFQI) to assess the central sensitivity to thyroid hormones in 2019 and found that thyroid hormones resistance was associated with increased risks of obesity, diabetes, and metabolic syndrome. They proposed a hypothesis that thyroid hormone resistance occurs in the body under certain circumstances, and thyroid hormone sensitivity decreases (5). Central thyroid hormone sensitivity indexes also include thyrotropin thyroxine resistance index (TT4RI) and TSH index (TSHI), which are also associated with many metabolic diseases (6).TT4RI, TSHI, and TFQI are introduced as quantitative markers for pituitary thyrotropic function (8, 9).

The ratio of free T3 to free T4 (FT3/FT4) is used to evaluate the rate of conversion of T4 to the more active T3 form, which reflects the sensitivity of peripheral tissues to thyroid hormones (7). Increasing attention has been paid to the relationship between thyroid hormone sensitivity and metabolic disorders in recent years, and sensitivity to thyroid hormone indices have been proved to be reliable predictors of insulin resistance, type 2 diabetes (T2D), and disorders of glucose and lipid metabolism (5, 8, 9). A study from the United States suggested that increased risk for all-cause mortality was associated with decreased central sensitivity to thyroid hormones. Furthermore, the FT3/FT4 ratio showed a U-shaped association with mortality (10).

Previous studies focused on patients with thyroid dysfunction, especially overt and/or subclinical hypothyroidism. The association between reduced sensitivity to thyroid hormones and ASCVD risk factors have not been well known. The association between impaired thyroid hormone sensitivity and ASCVD risk factors may help to understand the role of thyroid hormone in metabolic regulation, and provide theoretical basis for early intervention of lipid metabolism disorders and reduction of cardiovascular disease risk. In euthyroid individuals, subtle alterations in thyroid hormone sensitivity could impact cardiovascular health. In our study, we focused on these indices to explore their relationship with impaired sensitivity to thyroid hormones and ASCVD risk factors in a euthyroid population aiming to uncover potential associations that could inform clinical practice and future research.

## 2 Materials and methods

### 2.1 Study population

**Figure 1:**
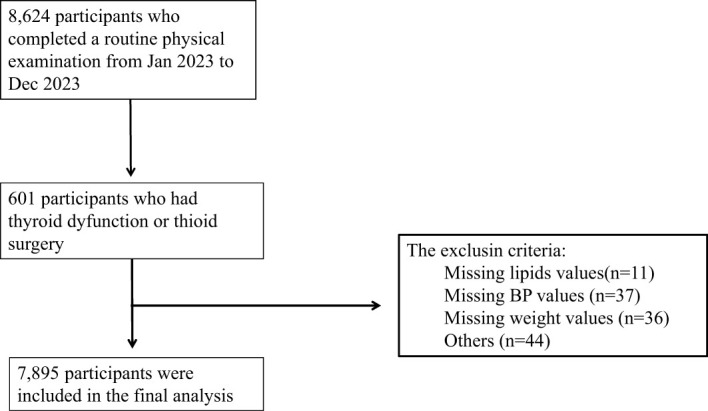
The flowchart of the selection of participants for the study.

This cross-sectional study enrolled participants (aged ≥ 18years) who underwent a routine physical examination from January 2023 to December 2023 in Strategic Support Force Medical Center. A total of 7,895 participants were included in this study, including 4,126 males and 3,769 females. The inclusion criteria were (1) with normal thyroid function; (2) aged ≥ 18years. The exclusion criteria were as follows: (1) incomplete data; (2) age younger than 18 years; (3) with known history of thyroid disease or thyroid surgery; (4) had been treated with drugs potentially altering thyroid hormone concentrations such as amiodarone and corticosteroids; (5) with history of pituitary disease; (6) with history of malignancy; (7) and pregnancy. Finally, 7,895 subjects were enrolled in this analysis (**Figure 1**).

All participants provided informed consent and the institutional ethics board approved the study of Ninth Medical Center of Chinese PLA General Hospital. This study adhered to the principles of the Declaration of Helsinki.

It is important to note that this study employs a cross-sectional design, which precludes the establishment of causality between the variables examined.

### 2.2 Laboratory measurements

Blood samples were drawn from all participants between 7:00 and 9:00 am after 8 to 10 hours of overnight fasting. Blood samples were centrifuged within 30 to 45 minutes of collection. Weight, height, and blood pressure were measured according to standard protocols by the same trained staff. TSH, FT4, and FT3 were evaluated by electrochemiluminescence immunoassay using an Abbott Architect i2000 ((Roche 801, Germany). Serum triglyceride (TG), total cholesterol (TC), high-density lipoprotein cholesterol (HDL-C), low-density lipoprotein cholesterol (LDL-C), were measured using the enzymatic colorimetric method with cholesterol esterase, cholesterol oxidase, and glycerol phosphate oxidase, respectively. All biochemical tests were performed on the day of sampling using commercial kits by the auto-analyzer ((Roche 801, Germany), and samples were analyzed only when quality control met the acceptable criteria.

### 2.3 Definition

Hypertension was defined as systolic blood pressure (SBP) ≥ 140 mmHg or diastolic blood pressure (DBP) ≥ 90mmHg, currently taking antihypertensive agents. Dyslipidemia was defined as TC ≥ 5.2 mmol/L or LDL-C ≥ 3.4 mmol/L or HDL-C < 1.0mmol/L or TG ≥ 1.7 mmol/L.

### 2.4 Calculation

The equations used for calculation are as follows:

TFQI = cdf FT4 − (1 − cdf TSH) (5); cdf: cumulative distribution function;

TSHI = ln TSH (mIU/L) + 0.1345 × FT4 (pmol/L) (11);

BMI=Weight (kg)/height(m)^2^

TyG = Ln[TG (mg/dL) × FPG (mg/dL)/2]

A higher value of TSHI or TT4RI indicates a lower central sensitivity to THs. As for TFQI, a negative value indicates greater pituitary sensitivity to THs, a positive value indicates less sensitivity and a value of 0 indicates normal sensitivity. TFQI was calculated with FT4 and TSH values according to the empirical cumulative distribution function. The advantage of TFQI is that it will not produce extreme values in the case of thyroid dysfunction and is more stable than TT4RI and TSHI. The TFQI value ranges from − 1 to 1. FT3 to FT4 ratio (FT3/FT4) was used to assess peripheral thyroid sensitivity. The higher the FT3/FT4 ratio, the better peripheral sensitivity to thyroid hormones (9). The triglyceride-glucose (TyG) index, determined from fasting plasma glucose (FPG) and triglycerides (TG), has been proposed as a convenient and reliable method to evaluate peripheral IR (12), as well as a potential risk marker of various disease (13, 14).

### 2.5 Statistic methods

In this analysis, continuous baseline variables were summarized using the mean ± standard deviation (SD) and compared using t-tests. The two-sided P value <0.05 was considered statistically significant. The enumeration data were expressed as frequencies and percentages (n [%]), and group comparisons were compared using one-way ANOVA test. The association between ASCVD risk factors and thyroid sensitivity was investigated using multivariable linear analyses. Multivariate logistic regression analysis was used to evaluate the relationship between thyroid hormone sensitivity and ASCVD risk factors. All data was analyzed using the statistical software SPSS 23.0, with a two-tailed p-value < 0.05 considered statistically significant.

## 3 Result

### 3.1 Baseline characteristic of the study population

The 7,895 euthyroid participants in the study cohort were18 years of age and older with a mean age of 43.93 years (range: 18–89 years). The proportion of females is highest in Q1 and decreases progressively from Q1 to Q4.With higher TFQI quartiles, the BMI of the population was lower (from 24.64kg/m² in the 1^st^ quartile to 24.20 kg/m² in the 4th quartile, p < 0.05; **Table 1**). However, the sUA levels were higher with progressively higher TFQI quartiles (from 333.80 ± 87.58 µmol/L in the1st quartile to 358.07 ± 92.97 µmol/L in the 4th quartile, p < 0.05; **Table 1**). With higher TFQI quartiles, the TC and TG of the population were progressively lower (TC: from 5.13mmol/L in 1^st^ quartile to 5.05 4^th^ quartile; TG: from1.75mmol/L in 1^st^ quartile to 1.60mmol/L in 4^th^ quartile, 1.60; P< 0.05; **Table 1**). With higher TFQI quartile, the DBP of the population was progressively higher (from 75.17mmHg in 1^st^ quartile to 77.25mmHg in 4^th^ quartile, p < 0.05; **Table 1**). **Table 1** presents the trends of age, blood lipids, blood pressure, uric acid, BMI, blood glucose, and thyroid hormones with increasing Thyroid Feedback Quantile Index (TFQI) quartiles among euthyroid participants.

**Table 1:** Baseline characteristic of the study population based on TFQI quartile (x ± SD).

Variables	Total population (n=7,895)	TFQI				P values
		1^st^ Quartile (Q1)	2^nd^ Quartile (Q2)	3^rd^ Quartile (Q3)	4^th^ Quartile (Q4)	
		-1,-0.264	-.0264,0.007	-0.007,0.235	0.235,1	
		(n=1995)	(n=2004)	(n=1927)	(n=1969)	
Age (year)	43.93 ± 12.82	46.16 ± 12.42	44.54 ± 12.30	43.34 ± 12.87	41.64 ± 13.20	0.000
Sex (M/F)	7895 (4126/3769)	1995 (994/1001)	2004 (1036/968)	1927 (1012/915)	1969 (1084/885)	0.00
BMI (kg/m2)	24.40 ± 3.64	24.64 ± 17.47	24.50 ± 3.51	24.23 ± 3.62	24.20 ± 3.71	0.000
SBP (mmHg)	123.44 ± 17.39	122.99 ± 17.47	122.72 ± 16.79	123.51 ± 18.10	124.46 ± 17.29	0.007
DBP (mmHg)	76.03 ± 10.62	75.17 ± 11.04	75.64 ± 10.58	76.02 ± 10.70	77.25 ± 10.08	0.000
LDL-C (mmol/L)	2.75 ± 0.76	2.76 ± 0.76	2.76 ± 0.76	2.75 ± 0.76	2.72 ± 0.77	0.182
HDL-C (mmol/L)	1.63 ± 0.38	1.59 ± 0.37	1.62 ± 0.38	1.64 ± 0.38	1.64 ± 0.37	0.000
TC (mmol/L)	5.10 ± 0.95	5.13 ± 0.94	5.13 ± 0.97	5.08 ± 0.92	5.05 ± 0.95	0.014
TG (mmol/L)	1.68 ± 1.32	1.75 ± 1.50	1.73 ± 1.46	1.64 ± 1.15	1.60 ± 1.15	0.014
FBG (mmol/L)	5.59 ± 1.05	5.54 ± 1.01	5.55 ± 0.93	5.63 ± 1.10	5.62 ± 1.11	0.000
UA (umol/L)	344.44 ± 89.62	333.80 ± 87.58	341.48 ± 87.44	344.30 ± 89.48	358.07 ± 92.97	0.000
TyG	1.02 ± 0.32	1.03 ± 0.33	1.03 ± 0.31	1.02 ± 0.31	1.01 ± 0.30	
TSH (mIu/l)	2.54 ± 2.14	1.56 ± 0.45	2.11 ± 0.73	2.87 ± 1.83	3.50 ± 1.53	0.000
FT3 (pmol/L)	5.11 ± 084	4.93 ± 0.65	5.07 ± 0.62	5.10 ± 0.64	5.28 ± 0.66	0.000
FT4 (pmol/L)	17.26 ± 2.81	15.51 ± 1.48	16.80 ± 1.89	17.56 ± 2.73	19.13 ± 2.67	0.000
TT3	1.82 ± 0.36	1.78 ± 0.31	1.80 ± 0.32	1.83 ± 0.35	1.87 ± 0.40	0.000
TT4	98.23 ± 18.05	91.11 ± 14.27	98.92 ± 18.47	99.48 ± 17.36	105.98 ± 19.91	0.000

BMI, body mass index; BP, blood pressure; HDL-C, high-density lipoprotein cholesterol; LDL-C, low-density lipoprotein cholesterol; TC, total cholesterol; TG, triglycerides; FBG, fasting blood glucose; TFQI, Thyroid Feedback Quantile-based Index; TyG, Triglyceride glucose index; TSH, Thyroid-stimulating hormone; fT4, free thyroxine; FT3, free triiodothyronine.

### 3.2 The prevalence of metabolic abnormalities at different TSQI quantiles

The research delves deeper, comparing the prevalence of ASCVD risk factors across the TFQI quartiles (**Table 2**). When specifically examining hyperuricemia, a crucial component of ASCDV, a statistical significance emerges when juxtaposing the four groups (p < 0.001, **Table 2**). However, in the case of lipid metabolism disorders, hyperglycemia, and hypertension, the data do not reveal any statistically significant differences across the four quartiles.

**Table 2:** Prevalence of metabolic abnormalities at different TSQI quantiles (%).

Variables	TFQI				*P* values
	Q1	Q2	Q3	Q4	
High BP	16.8	15.3	18.8	18.8	0.068
High BMI	54.8	57.0	55.5	54.2	0.541
High UA	16.9	18.4	19.7	22.7	0.000
High BG	16.1	15.1	17.4	15.3	0.337
High LDL	26.0	26.7	24.8	24.8	0.370
High HO	44.7	44.9	42.2	44.8	0.117
High TG	34.7	35.9	33.7	33.2	0.238
Low HDL	7.8	6.0%	6.9	7.0	0.008

### 3.3 The number of metabolic abnormalities at different TSQI quantiles

**Figure 2:**
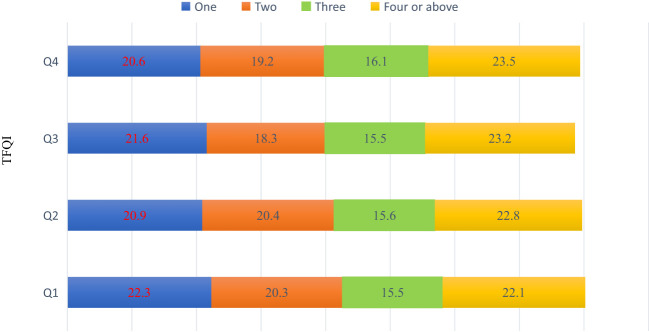
The number of metabolic abnormalities at different TSQI quantiles (%).

Another dimension of the research involves an in-depth exploration of the co-occurrence of various metabolic abnormalities across the four quartiles. Intriguingly, the data do not exhibit any statistically significant differences in the number of metabolic abnormalities across the quartiles, **Figure 2**.

### 3.4 Differences of thyroxine sensitivity indicators in different metabolic populations

We compared diverse thyroid hormone sensitivity indicators within populations grappling with different metabolic diseases (**Table 3**). Specifically, the F3/FT4 was found to exhibit statistical significance when juxtaposed across populations with high uric acid levels, high fasting blood sugar, and high triglycerides. Furthermore, the TSHI in populations with high TC levels was found to be higher than that in populations with normal TC levels (3.11 ± 0.55 vs. 3.14 ± 0.56, p < 0.05; **Table 3**).

**Table 3:** Differences of thyroxine sensitivity indicators in different metabolic populations.

Variables	Y or N	Ln TFQI	TSHI	FT3/FT4
HP	Y	0.82 ± 0.54	3.15 ± 0.56	0.30 ± 0.04
	N	0.80 ± 0.48	3.12 ± 0.56	0.30 ± 0.05
High UA	Y	0.81 ± 0.49	3.18 ± 0.55	0.29 ± 0.04
	N	0.80 ± 0.49	3.11 ± 0.55	0.31 ± 0.04^**^
Obesity	Y	0.79 ± 0.49	3.10 ± 0.58	0.30 ± 0.04
	N	0.81 ± 0.55	3.13 ± 0.55	0.32 ± 0.05^**^
High FBG	Y	0.81 ± 0.50	3.13 ± 0.53	0.30 ± 0.05
	N	0.81 ± 0.49	3.13 ± 0.56	0.31 ± 0.05^*^
High LDL	Y	0.81 ± 0.47	3.12 ± 0.55	0.30 ± 0.04
	N	0.80 ± 0.51	3.13 ± 0.55	0.30 ± 0.05
High TG	Y	0.81 ± 0.50	3.10 ± 0.53	0.29 ± 0.04
	N	0.81 ± 0.49	3.14 ± 0.57	0.31 ± 0.05^**^
Low HDL	Y	0.81 ± 0.54	3.13 ± 0.56	0.30 ± 0.04
	N	0.81 ± 0.49	3.02 ± 0.54	0.30 ± 0.04
High TC	Y	081 ± 0.50	3.11 ± 0.55	0.29 ± 0.05
	N	0.81 ± 0.48	3.14 ± 0.56^*^	0.30 ± 0.04

**P<0.01, *P<0.05.

### 3.5 Correlation between different thyroxine sensitivity indexes and risk factors for ASCVD

Another dimension of the research involves a systematic examination of the relationship between ASCVD risk factors and thyroid sensitivity (**Table 4**). The results revealed that TFQI positively correlates with DBP, SBP, TG, FBG, and UA, and the TyG index. On the other hand, TSHI positively correlates with SBP, DBP, FBG, UA, and the TyG index. Additionally, FT3/FT4 negatively correlates with DBP, SBP, TC, TG, and UA, while positively correlating with HDL-C and the TyG index.

**Table 4:** Correlation between different thyroxine sensitivity indexes and risk factors for ASCVD.

Variables	TFQI		TSHI		FT3/FT4	
	r	P values	r	P values	r	P values
DBP	0.087	0.000	0.071	0.071	-0.031	0.021
SBP	0.084	0.000	0.000	0.000	-0.034	0.012
LDL-C	0.002	0.856	0.007	0.628	-0.010	0.444
HDL-C	0.003	0.765	0.008	0.549	0.085	0.000
TC	0.007	0.628	0.005	0.739	-0.041	0.001
TG	0.026	0.049	-0.12	0.371	0.036	0.008
FBG	0.092	0.000	0.088	0.000	-0.23	0.088
UA	0.064	0.000	0.062	0.000	-0.028	0.041
TyG	0.029	0.041	0.035	0.011	0.070	0.000

### 3.6 Associations between TFQI and risk factors for ASCVD

The **Table 5** presents the odds ratios (OR) and 95% confidence intervals (CI) for ASCVD risk factors across four quartiles (Q1, Q2, Q3, Q4), with Q1 serving as the reference group. The odds of high UA increase significantly in Q4 (OR = 1.21, P = 0.03), indicating a potential positive association with higher quartiles. The odds of obesity are significantly lower in Q3 and Q4 (OR = 0.81, P=0.023 and P=0.024, respectively), suggesting a negative association with higher quartiles. HP is a strong positive association with increasing quartiles, especially in Q3 (OR = 1.40, P < 0.001) and Q4 (OR = 1.50, P < 0.001). The odds of high FBG are significantly higher in Q3 (OR = 1.28, P = 0.007), but not in Q4.High LDL, High TG, and High TC: No significant associations were observed across the quartiles for these indicators, indicating relatively stable odds, but the results for Low HDL (Low High-Density Lipoprotein) show a significant association across the quartiles.

**Table 5:** Associations between TFQI and risk factors for ASCVD.

Variables	OR (95%CI)	TFQI			
		Q1	Q2	Q3	Q4
High UA	OR (95%CI)	1 (Reference)	1.02 (0.85, 1.22)	1.18 (0,98, 1.40)	1.21 (1.02, 1.44)
	P value		0.85	0.08	0.03
Obesity	OR (95%CI)	1 (Reference)	0.87 (0.73, 1.04)	0.81 (0.68, 0.97)	0.81 (0.67, 0.97)
	P value		0.12	0.023	0.024
HP	OR (95%CI)	1 (Reference)	0.97 (0.81,1.17)	1.40 (1.17,1.68)	1.50 (1.25,1.79)
	P value		0.778	0.00	0.00
High FBG	OR (95%CI)	1 (Reference)	1.00 (0.83,1.20)	1.28 (1.07,1.53)	1.13 (0.94,1.36)
	P value		0.97	0.007	0.194
High LDL	OR (95%CI)	1 (Reference)	1.07 (0.92,1.23)	0.99 (0.86,1.15)	1.01 (0.87,1.17)
	P value		0.39	0.92	0.94
High TG	OR (95%CI)	1 (Reference)	1.06 (0.93,1.21)	1.01 (0.88,1.16)	0.82 (0.72,0.95)
	P value		0.38	0.88	0.08
Low HDL	OR (95%CI)	1 (Reference)	0,78 (0.61,1.00)	0.61 (0.47,0.80)	0.62 (0.47,0.81)
	P value		0.00	0.00	0.00
High TC	OR (95%CI)	1 (Reference)	1.06 (0.93,1.20)	0.98 (0.86,1.11)	0.96 (0.84,1.10)
	P value		0.40	0.73	0.58

ALL analysis adjust for age and sex.

## 4 Discussion

Previous studies have only analyzed the correlation between thyroid hormone sensitivity and individual metabolic indicators in euthyroid populations. This study is the first to comprehensively analyze the relationship between thyroid hormone sensitivity and various risk factors for atherosclerotic cardiovascular disease (ASCVD), as well as the association between the number of metabolic abnormalities and thyroid hormone sensitivity. The present study provides novel insights into the relationship between thyroid hormone sensitivity and the risk factors for ASCVD in a euthyroid population in Beijing. The findings suggest that even within the normal range of thyroid function, subtle changes in hormone sensitivity could impact cardiovascular health, potentially contributing to ASCVD development. We provide evidence of the association between sensitivity to thyroid hormones and metabolic disorders, including obesity, dyslipidemia, blood pressure, TyG, HUA and high FBG. Using central and peripheral thyroid hormone sensitivity, we evaluated thyroid hormone sensitivity and associations with ASCVD risk factors in the euthyroid population.

Impaired thyroid hormone sensitivity has been reported in various euthyroid populations, with several studies highlighting its association with adverse health outcomes. A study by Mehran et al. (15)found that reduced sensitivity to thyroid hormone was associated with diabetes and hypertension in euthyroid individuals. Similarly, Sun et al. (16) reported that impaired thyroid hormone sensitivity was linked to hyperuricemia, obesity, and cardiovascular disease risk in subjects with subclinical hypothyroidism. These findings suggest that thyroid hormone sensitivity indices may serve as early indicators of metabolic and cardiovascular risks in euthyroid populations.

Impaired thyroid hormone sensitivity in euthyroid individuals has significant clinical implications, particularly in terms of metabolic and cardiovascular health. A study by Ye et al. (17) found that euthyroid participants with impaired thyroid hormone sensitivity, as indicated by higher TSHI, TT4RI, TFQI, and PTFQI values, had a lower heart rate (≤60 beats/min). This suggests that impaired thyroid hormone sensitivity may be associated with bradycardia, which could potentially impact cardiovascular function.

We observed that a higher proportion of males are found in the upper quartiles of TFQI suggests that males may have greater thyroid hormone sensitivity compared to females. This may be attributed to the fact that males typically exhibit higher testosterone levels, which have the capacity to augment thyroid hormone sensitivity and metabolism, thereby potentially elevating TFQI values. Additionally, the higher basal metabolic rates observed in males may further enhance thyroid hormone sensitivity, consequently resulting in higher TFQI values.

Thyroid hormone sensitivity indices may also be associated with body composition and metabolic syndrome. A study by Ying Li et al. (18)found that impaired thyroid hormone sensitivity was associated with an increased body fat mass/muscle mass ratio (F/M) in euthyroid individuals, particularly in males, individuals under 65 years of age, and those with a BMI ≥ 25 kg/m². This association highlights the potential role of thyroid hormone sensitivity in modulating body composition and metabolic health.

Our findings reveal a significant association between impaired sensitivity to thyroid hormones and several ASCVD risk factors, including hypertension, dyslipidemia, and high uric acid. These relationships underscore the multifaceted nature of ASCVD pathogenesis and suggest that thyroid hormone sensitivity could be an underappreciated factor in cardiovascular risk stratification.

Euthyroid subjects with higher TFQI in the current study were less prone to have a high BMI and high LDL in contrast to previous reports (5, 19), but consistent with Mehran’s findings (15). TFQI is mainly an indicator of central resistance to thyroid hormones. Higher FT4 levels in patients with reduced sensitivity to thyroid hormones results in an elevated muscle-derived resting energy expenditure and TH-mediated mitochondrial uncoupling, which protects excess weight gain. An individual with high central thyroid hormone sensitivity, that is, TSH has a better response to T4. Therefore, it seems rational to have a higher BMI in individuals with high thyroid hormone sensitivity, which is not consistent with our results. We speculate that this discrepancy reflects the characteristics of sensitivity and metabolism in euthyroid individuals, and peripheral thyroid sensitivity regulation may play a role in this.

MITCHELL et al. reported RTH has positively correlated with SBP and DBP (20). In the current survey, SBP and DBP was consistently associated with all indices of RTH, such as TSHI, and TFQI, in euthyroid subjects, with higher TFQI quartile, the DBP of the population was progressively higher, the strong positive association between HP and increasing quartiles, particularly in Q3 (OR = 1.40, P < 0.001) and Q4 (OR = 1.50, P < 0.001). This finding aligns with previous studies linking chronic infections to systemic inflammation and cardiovascular risk. Given the high prevalence of HP infection in many populations, screening and treatment for HP could potentially reduce ASCVD risk which might justify the reduced effect of thyroid hormones in arterial smooth muscle relaxation leading to increased systemic vascular resistance and reduced endothelium-dependent vasodilatation and nitricoxide availability.

TSHI and TFQI were differently associated with adverse metabolic outcomes in the whole population, which might be derived by the extreme values of TSH and fT4 in the total population. TFQI was significantly associated with most ASCVD risk factors except for LDL-C, HDL-C and TC in the whole population. The absence of significant associations for high LDL, high TG, and high TC across the quartiles suggests that these lipid parameters may not be strongly influenced by the quartile levels in this study. The relatively stable odds across quartiles could be due to effective lipid management or the influence of other confounding variables. TSHI was associated with SBP, FBG, UA and TyG, FT3/F4 was also significantly associated with most ASCVD risk factors except for FBG and LDL-C. The different results found for various indices may be related to the formula defined for each index. In TFQI, both parameters of TSH and fT4 are incorporated in calculating resistance to thyroid hormones, and these indices present the joint distribution of both fT4 and TSH values with the advantage of not yielding extreme values of thyroid hormones. TSHI represent extreme values of each FT4.

While our analysis revealed statistically significant differences in HDL-C and BMI across quartiles (p < 0.000), the magnitude of these differences was small. These findings highlight the importance of considering both statistical significance and effect size when interpreting results. In contrast, variables such as TFQI, TSH, and FT3/FT4 showed larger effect sizes and more clinically relevant differences, emphasizing the need for a balanced interpretation of the data.

Previous studies reported a high incidence of HUA in patients with hypothyroidism and hyperthyroidism (21, 22); in patients with hyperthyroidism, elevated serum urate is closely correlated with T4 concentration (23). Other studies have found that the relationship between TSH levels and serum urate is weak, or that hypothyroidism is closely related to gout, but is weakly related to HUA (24). In our study, we found that euthyroid individuals had an increase in uric acid as thyroid hormone sensitivity decreases. Uric acid were positively correlated with central thyroid hormone resistance and negatively correlated with peripheral thyroid hormone sensitivity. The significant increase in the odds of high UA in the highest quartile (Q4, OR = 1.21, P = 0.024) suggests a potential positive association with higher quartile levels. Elevated uric acid levels have been implicated in cardiovascular risk through mechanisms such as endothelial dysfunction and inflammation. This finding underscores the importance of monitoring uric acid levels, especially in individuals with other cardiovascular risk factors.

Diabetes and thyroid disease are two closely related disorders. Some studies reported a positive correlation between FT4 and FPG levels (25). Regarding the relationship between blood glucose and thyroid hormone sensitivity, our study found decreased thyroid hormone sensitivity to be associated with increased FBG. The significant increase in the odds of high FBG in Q3 (OR = 1.28, P = 0.007) but not in Q4 highlights the complex relationship between fasting blood glucose and cardiovascular risk. The lack of significance in Q4 might be due to a plateau effect or differences in the distribution of other risk factors. This finding emphasizes the need for early detection and management of hyperglycemia. This result may explain the discordant findings previously reported. These associations were statistically significant results in TSHI, TFQI and FT3/FT4. Several cross-sectional studies among Chinese population reported that a decreased sensitivity/increased resistance to thyroid hormones were associated with reduced risk of pre-diabetes or BMI (26, 27). SCH is a strong indicator of risk for atherosclerosis and myocardial infarction in older women (3). For patients with hypothyroidism or SCH with low T4 sensitivity and preexisting CVD, it is worth exploring whether appropriate therapeutic intervention is necessary. Because our analysis focused on sensitivity to thyroid hormone indices, which are all FT4 based, FT3 was intentionally excluded from the models, since it was considered to be downstream in the action pathway. Actually, deiodinases balance may play a role in the modulation of sensitivity to thyroid hormones (28), and future projects should be devoted to specifically study their role in resistance to thyroid hormone in obesity, metabolic syndrome, and diabetes.

The current study has several strengths, including the large sample size and population-based design, the novelty value of the subject, suitable exclusion criteria, adjusting for relevant confounding factors, and assessment of 3 different indices of thyroid hormone resistance in both the euthyroid and total population.

### 4.1 Limitations

Several limitations in this study should be acknowledged. First, current data were from only one hospital. The findings should be confirmed in other populations. Second, as with other observational designs, the causal relationship might not be inferred, and potential residual confounding was unavoidable, although we have adjusted for the major confounders. Third, The cross-sectional nature of this study is a significant limitation, as it restricts our ability to draw definitive causal inferences from the observed associations. Future research should consider longitudinal designs to better understand the temporal relationships between the variables examined in this study.

## 5 Conclusions

This study provides evidence that impaired sensitivity to thyroid hormones is associated with ASCVD risk factors in a euthyroid population in Beijing. Therefore, we hypothesized that RTH could be used as a new predictor of ASCVD providing help for early screening and preventive of ASCVD. The findings underscore the need for further research to understand the mechanisms underlying this association and to explore potential therapeutic interventions.

In conclusion, this study provides evidence that impaired sensitivity to thyroid hormones is associated with ASCVD risk factors in a euthyroid population. These findings contribute to our understanding of the complex interplay between thyroid function, thyroid hormone sensitivity, and cardiovascular health. They also highlight the need for further research to explore the potential clinical utility of assessing thyroid hormone sensitivity in cardiovascular risk stratification and management. Future research should explore these associations using longitudinal or experimental designs to establish causality and provide a more comprehensive understanding of the underlying mechanisms.

## Data Availability

Due to ethical considerations, the database of this study cannot be publicly accessible. Requests to access these datasets should be directed to 1270802646@qq.com.

## References

[1] Kim B. Thyroid hormone as a determinant of energy expenditure and the basal metabolic rate. Thyroid. (2008) 18:141–4. doi: 10.1089/thy.2007.0266, PMID: 18279014

[2] Floriani C Gencer B Collet TH Rodondi N. Subclinical thyroid dysfunction and cardiovascular diseases: 2016 update. Eur Heart J. (2018) 39:503–7. doi: 10.1093/eurheartj/ehx050, PMID: 28329380

[3] Hak AE Pols HA Visser TJ Drexhage HA Hofman A Witteman JC. Subclinical hypothyroidism is an independent risk factor for atherosclerosis and myocardial infarction in elderly women: the Rotterdam Study. Ann Intern Med. (2000) 132:270–8. doi: 10.7326/0003-4819-132-4-200002150-00004, PMID: 10681281

[4] Zhang X Chen Y Ye H Luo Z Li J Chen Z. Correlation between thyroid function, sensitivity to thyroid hormones and metabolic dysfunction-associated fatty liver disease in euthyroid subjects with newly diagnosed type 2 diabetes. . Endocrine. (2023) 80:366–79. doi: 10.1007/s12020-022-03279-2, PMID: 36539681

[5] Laclaustra M Moreno-Franco B Lou-Bonafonte JM Mateo-Gallego R Casasnovas JA Guallar-Castillon P. Impaired sensitivity to thyroid hormones is associated with diabetes and metabolic syndrome. Diabetes Care. (2019) 42:303–10. doi: 10.2337/dc18-1410, PMID: 30552134

[6] Corica D Licenziati MR Calcaterra V Curro M Di Mento C Curatola S. Aversa T et al: Central and peripheral sensitivity to thyroid hormones and glucose metabolism in prepubertal children with obesity: pilot multicenter evaluation. Endocrine. (2023) 80:308–11. doi: 10.1007/s12020-022-03276-5, PMID: 36484935

[7] Ma D Zhao P Gao J Guo X Han M Zan X. The correlation between impaired thyroid hormone sensitivity and diabetic nephropathy in euthyroid patients with type 2 diabetes mellitus. Diabetes Metab Syndr Obes. (2025) 18:1207–21. doi: 10.2147/DMSO.S507750, PMID: 40291540 PMC12034277

[8] Liu ZM Li G Wu Y Zhang D Zhang S Hao YT. Xie Y et al: Increased Central and Peripheral Thyroid Resistance Indices During the First Half of Gestation Were Associated With Lowered Risk of Gestational Diabetes-Analyses Based on Huizhou Birth Cohort in South China. Front Endocrinol (Lausanne). (2022) 13:806256. doi: 10.3389/fendo.2022.806256, PMID: 35345468 PMC8957094

[9] Stepanek L Horakova D Stepanek L Janout V Janoutova J Bouchalova K. Free triiodothyronine/free thyroxine (FT3/FT4) ratio is strongly associated with insulin resistance in euthyroid and hypothyroid adults: a cross-sectional study. Endokrynol Pol. (2021) 72:8–13. doi: 10.5603/EP.a2020.0066, PMID: 33125689

[10] Yu G Liu S Song C Ma Q Chen X Jiang Y. Association of sensitivity to thyroid hormones with all-cause mortality in euthyroid US adults: A nationwide cohort study. Eur Thyroid J. (2024) 13. doi: 10.1530/ETJ-23-0130, PMID: 38189656 PMC10895331

[11] Jostel A Ryder WD Shalet SM. The use of thyroid function tests in the diagnosis of hypopituitarism: definition and evaluation of the TSH Index. Clin Endocrinol (Oxf). (2009) 71:529–34. doi: 10.1111/j.1365-2265.2009.03534.x, PMID: 19226261

[12] Li Z He Y Wang S Li L Yang R Liu Y. Zheng H et al: Association between triglyceride glucose index and carotid artery plaque in different glucose metabolic states in patients with coronary heart disease: a RCSCD-TCM study in China. Cardiovasc Diabetol. (2022) 21:38. doi: 10.1186/s12933-022-01470-3, PMID: 35277186 PMC8917731

[13] Yan F Yan S Wang J Cui Y Chen F Fang F. Association between triglyceride glucose index and risk of cerebrovascular disease: systematic review and meta-analysis. Cardiovasc Diabetol. (2022) 21:226. doi: 10.1186/s12933-022-01664-9, PMID: 36324146 PMC9632026

[14] Park B Lee HS Lee YJ. Triglyceride glucose (TyG) index as a predictor of incident type 2 diabetes among nonobese adults: a 12-year longitudinal study of the Korean Genome and Epidemiology Study cohort. Transl Res. (2021) 228:42–51. doi: 10.1016/j.trsl.2020.08.003, PMID: 32827706

[15] Mehran L Delbari N Amouzegar A Hasheminia M Tohidi M Azizi F. Reduced sensitivity to thyroid hormone is associated with diabetes and hypertension. J Clin Endocrinol Metab. (2022) 107:167–76. doi: 10.1210/clinem/dgab646, PMID: 34480566

[16] Sun Y Teng D Zhao L Shi X Li Y Shan Z. Impaired sensitivity to thyroid hormones is associated with hyperuricemia, obesity, and cardiovascular disease risk in subjects with subclinical hypothyroidism. Thyroid. (2022) 32:376–84. doi: 10.1089/thy.2021.0500, PMID: 35078326

[17] Ye G Zhang Y Peng L Yu Z Bai Y Wu M. : Impaired sensitivity to thyroid hormones is associated with lower heart rate in the euthyroid population. Heart Rhythm. (2025). doi: 10.1016/j.hrthm.2025.02.028, PMID: 39986552

[18] Li Y Zhang Q Chen L Wang Y Ye Q Liu W. Impaired sensitivity to thyroid hormones is associated with increased body fat mass/muscle mass ratio (F/M) in the euthyroid population. Diabetol Metab Syndr. (2025) 17:128. doi: 10.1186/s13098-025-01693-w, PMID: 40234912 PMC12001440

[19] Gokosmanoglu F Aksoy E Onmez A Ergenc H Topkaya S. Thyroid homeostasis after bariatric surgery in obese cases. Obes Surg. (2020) 30:274–8. doi: 10.1007/s11695-019-04151-5, PMID: 31617112

[20] Mitchell CS Savage DB Dufour S Schoenmakers N Murgatroyd P Befroy D. Curran S et al: Resistance to thyroid hormone is associated with raised energy expenditure, muscle mitochondrial uncoupling, and hyperphagia. J Clin Invest. (2010) 120:1345–54. doi: 10.1172/JCI38793, PMID: 20237409 PMC2846038

[21] Giordano N Santacroce C Mattii G Geraci S Amendola A Gennari C. Hyperuricemia and gout in thyroid endocrine disorders. Clin Exp Rheumatol. (2001) 19:661–5., PMID: 11791637

[22] Sato A Shirota T Shinoda T Komiya I Aizawa T Takemura Y. Hyperuricemia in patients with hyperthyroidism due to Graves’ disease. Metabolism. (1995) 44:207–11. doi: 10.1016/0026-0495(95)90266-X, PMID: 7869917

[23] Saini V Yadav A Arora MK Arora S Singh R Bhattacharjee J. Correlation of creatinine with TSH levels in overt hypothyroidism - a requirement for monitoring of renal function in hypothyroid patients? Clin Biochem. (2012) 45:212–4. doi: 10.1016/j.clinbiochem.2011.10.012, PMID: 22061337

[24] See LC Kuo CF Yu KH Luo SF Chou IJ Ko YS. Hyperthyroid and hypothyroid status was strongly associated with gout and weakly associated with hyperuricaemia. PloS One. (2014) 9:e114579. doi: 10.1371/journal.pone.0114579, PMID: 25486420 PMC4259336

[25] Gu Y Li H Bao X Zhang Q Liu L Meng G. Xia Y et al: The Relationship Between Thyroid Function and the Prevalence of Type 2 Diabetes Mellitus in Euthyroid Subjects. J Clin Endocrinol Metab. (2017) 102:434–42. doi: 10.1210/jc.2016-2965, PMID: 27906594

[26] Liu B Wang Z Fu J Guan H Lyu Z Wang W. Sensitivity to thyroid hormones and risk of prediabetes: A cross-sectional study. Front Endocrinol (Lausanne). (2021) 12:657114. doi: 10.3389/fendo.2021.657114, PMID: 34017311 PMC8129566

[27] Nie X Ma X Xu Y Shen Y Wang Y Bao Y. Increased serum adipocyte fatty acid-binding protein levels are associated with decreased sensitivity to thyroid hormones in the euthyroid population. Thyroid. (2020) 30:1718–23. doi: 10.1089/thy.2020.0011, PMID: 32394790

[28] Gereben B Zavacki AM Ribich S Kim BW Huang SA Simonides WS. Cellular and molecular basis of deiodinase-regulated thyroid hormone signaling. Endocr Rev. (2008) 29:898–938. doi: 10.1210/er.2008-0019, PMID: 18815314 PMC2647704

